# Misleading clinical and imaging features in atypical aggressive angiomyxoma of the female vulvovaginal or perianal region: report of three cases and review of the literature

**DOI:** 10.3389/fonc.2024.1373607

**Published:** 2024-03-25

**Authors:** Ling Zhang, Rong Liu, Jian Peng

**Affiliations:** ^1^Department of Obstetrics and Gynecology, National Clinical Research Center for Obstetrics and Gynecology, Tongji Hospital, Tongji Medical College, Huazhong University of Science and Technology, Wuhan, China; ^2^Key Laboratory of Cancer Invasion and Metastasis (Ministry of Education), Hubei Key Laboratory of Tumor Invasion and Metastasis, Tongji Hospital, Tongji Medical College, Huazhong University of Science and Technology, Wuhan, China; ^3^Department of Radiology, Tongji Hospital, Tongji Medical College, Huazhong University of Science and Technology, Wuhan, China

**Keywords:** aggressive angiomyxoma, perineal soft tissue mass, vestibular gland cyst, vaginal wall prolapse, ultrasonography, magnetic resonance imaging

## Abstract

**Objectives:**

Aggressive (deep) angiomyxoma (AAM) is a rare mesenchymal tumor that typically originates from the vulvovaginal region, perineum, and pelvis in adult women. The objective of this case report and literature review is to comprehensively analyze the clinical, imaging, and pathological characteristics of atypical AAM in the female lower genital tract and pelvic floor in order to minimize preoperative misdiagnosis or missed diagnosis and ultimately optimize the clinical management strategy.

**Methods:**

The data of three cases with atypical AAM, which demonstrate similarities with other lesions observed in the female lower genital tract over the past 1.5 years, were retrospectively described. This description included clinical management, images and reports of ultrasonography (US) and magnetic resonance imaging (MRI), clinicopathological features, follow-up, and outcomes. In the Discussion section, a review of the literature on MEDLINE (PubMed) and Web of Science from the past 50 years was conducted.

**Results:**

The three cases all underwent preoperative ultrasonography, and two of them also underwent preoperative MRI examination. Complete resection of the lesions was performed in all three cases, followed by postoperative pathological examination. The histopathology of these three cases revealed invasive angiomyxoma, as confirmed by immunohistochemical staining, which demonstrated positive expression of desmin, vimentin, estrogen, and progesterone receptors. The patients experienced a smooth postoperative recovery. Ultrasound had a diagnostic accuracy rate of 100% (3/3) for locating and determining the extent of the lesions; however, its specific diagnostic accuracy rate for identifying the pathological type was only 33% (1/3). In contrast, MRI had a diagnostic accuracy rate of 100% (2/2) for locating and determining the extent of lesions but did not show any specific diagnostic accuracy for identifying the pathological types.

**Conclusions:**

Our findings indicate that even if a vulvovaginal lesion presents with a superficial location, small size, limited scope, and regular shape, suspicion of atypical AAM should arise when palpation reveals toughness, tensility, and deformability under pressure. US reveals a well-defined hypoechoic to anechoic mass with uniformly distributed coarse dot echoes, with or without detectable intratumoral blood flow signal. MRI shows prolonged T1 and T2 signals with inhomogeneous enhancement and evident diffusion restriction on diffusion-weighted imaging (DWI).

## 1 Introduction

Aggressive (deep) angiomyxoma (AAM) is a rare, benign mesenchymal tumor that almost exclusively occurs in the deep soft tissues of the genital area. It is considered aggressive due to its local infiltration and high rate of local recurrence after resection (1, 2). The latest edition of the World Health Organization Classification of Tumors of Soft Tissue and Bone defines deep angiomyxoma as a uniformly paucicellular myxoedematous tumor with infiltrative margins and a prominent vascular component (3). To our knowledge, since AAM was first described in 1983 by Steeper and Rosai, approximately 400 cases have been reported in the literature (1). The reported female-to-male ratio ranges from 6.6:1 to 9:1 (4, 5).

Due to its rare occurrence and non-specific clinical manifestations, clinical misdiagnosis is common, and imaging is helpful for the clinical management of AAM and follow-up. Based on typical imaging features, the radiologist was able to suspect that the lesion represented AAM initially. If there are no specific signs indicating AAM presence, this lesion might be mistaken for an AAM mimic like vestibular gland cysts, leiomyomas, inflammation, or other soft tissue tumors. Surgical resection remains a primary treatment option. Accurate evaluation of both the type and extent of this lesion plays a vital role in determining an appropriate surgical approach for tumor removal. Ultimately, pathological diagnosis confirms whether it is indeed an instance of AAM.

We conducted a retrospective study of three patients with AAM to assess the atypical clinical and imaging features that may lead to misdiagnosis. To our knowledge, there is a lack of literature regarding AAM, which often presents challenges for accurate diagnosis. We thoroughly reviewed all available images, including ultrasonography (US) and magnetic resonance imaging (MRI). Our aim was to provide a comprehensive description of radiological features in conjunction with clinical and histological findings, ultimately reducing preoperative misdiagnosis or missed diagnoses.

## 2 Materials and methods

### 2.1 Study design

This study is a retrospective and descriptive analysis of the data collected in routine medical records of three patients with AAM, including the clinical management, US and MRI images and reports, clinicopathological features, follow-up, and outcomes. In the Discussion section, a review of the literature on MEDLINE (PubMed) and Web of Science from the past 50 years was conducted.

### 2.2 Study population

These three cases were all referred to the Department of Obstetrics and Gynecology of Tongji Hospital, affiliated with Tongji Medical College of Huazhong University of Science and Technology, from other hospitals between February 2022 and August 2023. In the hospital, all patients underwent detailed preoperative imaging examination, surgical and pathological diagnosis, and regular postoperative follow-up. Clinical demographic information, gynecological examination, surgical data, follow-up information, patient clinicopathological features, and imaging features were retrieved from the original electronic hospital medical records and image reporting system.

### 2.3 Statistical analyses

The statistical software package SPSS26.0 (IBM SPSS Statistics for Windows, 26.0 version, Armonk, NY, USA) was used for data analysis. The classified variables are represented as n (%).

## 3 Results

### 3.1 Case 1

#### 3.1.1 Clinical history

The patient, a 49-year-old woman, reported that she had been diagnosed with vaginal prolapse approximately 1 year ago. She experienced worsening symptoms when standing or being active, accompanied by urinary incontinence that worsened when sneezing. However, she did not have difficulty urinating and also experienced pain during urination. Initially, the patient received pelvic floor rehabilitation training for anterior vaginal wall prolapse at a local hospital; however, her symptoms did not improve. Subsequently, she was transferred to our department for further evaluation. Clinical gynecological examination revealed a multiparous and gravid vulva with a smooth vaginal canal and minimal secretions. During the Valsalva maneuver, there was an evident protrusion of the anterior vaginal wall extending beyond 3.0–4.0 cm of the hymen along with urinary retention. Mild cervical discharge was observed along with an anteverted uterus and no apparent abnormalities in the bilateral adnexa. The preliminary clinical diagnosis remained as anterior vaginal wall prolapse.

#### 3.1.2 Imaging examinations

**Figure 1:**
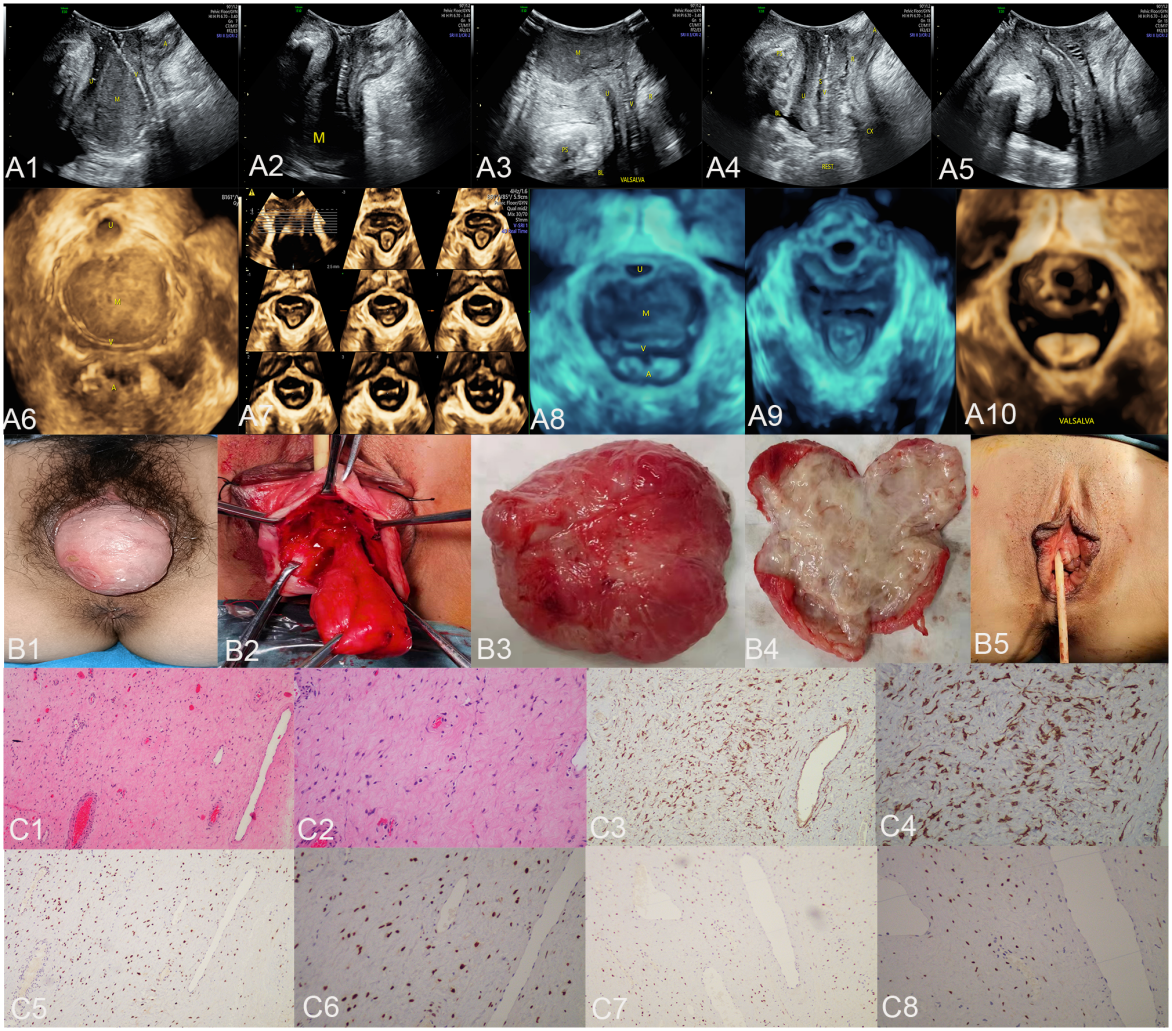
The ultrasound **(A1–A10)**, surgical **(B1–B5)**, and pathological images **(C1–C8)** of case 1. US: panels **(A1–A3)** respectively display the median sagittal sections of the transperineal ultrasound in the resting state, maximum contraction state, and maximum Valsalva state of the patient in the lithotomy position preoperatively. In panel **(A1)**, an AAM lesion is observed within the urethrovaginal space; it ascends to the posterior aspect of the bladder in panel **(A2)** and descends to the vaginal opening in panel **(A3)** (M represents the AAM lesion). All the lesions appeared as homogeneous hypoechoic masses with well-defined margins. Panels **(A4, A5)** show the median sagittal sections of perineal ultrasound in the resting state and maximum Valsalva state, respectively, of the patient 7 months post-surgery without any observed lesions. Panels **(A6, A8)** display the axial planes of HLAM reconstructed by three-dimensional ultrasound in the resting state preoperatively and during the Valsalva maneuver. Panel **(A7)** displays the axial plane of TUI during the maximum contraction state preoperatively. These axial planes provide information about the size, shape, and location of the AAM lesions. In panel **(A6)**, the lesion is observed protruding posteriorly from behind the urethra toward the vagina; in panel **(A7)**, the lesion has ascended completely above the HLAM; in panel **(A8)**, during the Valsalva maneuver, the lesion descends through the HLAM (M represents an AAM lesion). Panels **(A9, A10)** demonstrate the axial plane of the HLAM in the resting state and maximum Valsalva state, respectively, 7 months postsurgery for patient without any observed lesions. Surgery: panels **(B1, B2)** depict the lesion as an oval-shaped mass protruding from the anterior vaginal wall into the vaginal cavity. Panel **(B3)** displays the completely resected lesion, which is a soft tissue mass measuring 4.0 × 5.0 cm and exhibiting an oval shape with a pink appearance. Panel **(B4)** displays the lesion upon sectioning, which appears as a gray color with medium soft tissue consistency. Panel **(B5)** depicts the appearance of the vulva after the procedure has been completed. HE: Panel **(C1)**, at low magnification, shows that the tumor boundary appears indistinct, revealing a myxedema matrix interspersed with thin collagen fibers and blood vessels of varying thickness within the tumor. The tumor exhibits areas of loose and dense cellularity, with spindle cells uniformly scattered in dense regions without apparent organization (HE, ×100). As shown in panel **(C2)**, at high magnification, the tumor cells exhibited a low density and assumed a stellate or fusiform morphology without evident pleomorphism (HE, ×200). IHC: Tumor cells exhibited strong positive staining for desmin in panel **(C3)** (×100) and panel **(C4)** (×200), estrogen receptors in panel **(C5)** (×100) and panel **(C6)** (×200), and progesterone receptors in panel **(C7)** (×100) and panel **(C8)** (×200). US, ultrasonography; U, urethra; M, mass; V, vagina; A, anus; HLAM, hiatus of levator ani muscle; HE, histopathological examination; IHC, immunohistochemistry; AAM, aggressive angiomyxoma; TUI, tomographic ultrasound imaging.

The transperineal pelvic floor ultrasound revealed a hypoechoic mass measuring 3.9 × 3.6 × 4.6 cm, which was located in the space between the urethra and the middle-lower segment of the vagina. The mass had clear boundaries and showed obvious protrusion toward the vagina (**Figures 1A1–10**). Blood flow signals were observed surrounding it with a resistance index (RI) of 0.64. Ultrasonographic diagnosis suggested that the solid mass in the urethrovaginal space originated from the vaginal wall and was a leiomyoma. The patient did not undergo an MRI examination.

#### 3.1.3 Surgical findings

During the surgery, a palpable mass measuring approximately 4.0 × 5.0 cm in size was observed on the anterior vaginal wall under anesthesia, indicating evident protrusion (**Figure 1B1**). Notably, there was no discernible prolapse observed in the cervix or posterior vaginal wall. A longitudinal incision of approximately 1.0 cm was made in the mucosa and submucosal connective tissue of the anterior vaginal wall, located approximately 0.5 cm below the transverse sulcus of the urethra. A mass in the urethral space (**Figure 1B2**) was carefully dissected and completely excised, followed by transvaginal tension-free transobturator suburethral tape (TVT-O) surgery to suspend the middle part of the urethra. The excised tissue, measuring 4.0 × 5.0 cm and displaying an oval shape with a pink hue (**Figure 1B3**), exhibited a gray color and medium soft tissue consistency upon sectioning (**Figure 1B4**). Subsequently, it was sent for pathological examination. The vulva returned to its normal appearance after the surgical procedure (**Figure 1B5**).

#### 3.1.4 Pathological examination results

Histopathological examination of the specimen revealed a tumor composed of fibroblasts and maternal muscle fiber cells, exhibiting characteristics consistent with a benign-intermediate type. Microscopically, mild cellular morphology was observed in the spindle cell proliferation against a background of extensive mucus degeneration; irregular hyperplasia of small vessels in the stroma was also observed (**Figures 1C1,2**). Immunohistochemical analysis of the specimen demonstrated strong positive staining for desmin (**Figures 1C3,4**), estrogen receptor (ER) (**Figures 1C5,6**), and progesterone receptor (PR) (**Figures 1C7,8**), as well as smooth muscle actin (SMA), caldesmon, and CD10. Partial positivity was observed for S-100, while negative staining was observed for CD34, β-catenin, and myogenin. The Ki-67 immunohistochemical index was estimated to be approximately 1%. No HMGA2 rearrangement, CTNNB1 gene mutations, or APC gene mutations were detected by molecular analysis. The final histopathological diagnosis indicated AAM with positive surgical margins.

#### 3.1.5 Follow−up and outcomes

The patient reported no postoperative discomfort, and a postoperative ultrasound examination of the pelvic floor revealed no lesions. Therefore, there was no evidence of recurrence during the 1-month and 7-month follow-up visits.

### 3.2 Case 2

#### 3.2.1 Clinical history

The patient was a 53-year-old woman who presented with a tumor in the left perineum that had persisted for 6 months without pain, fever, or other discomfort. She experienced urine leakage when coughing and had not received any related treatment. Clinical examination revealed a married and delivered vulva, as well as a cystic solid mass that could be observed and touched in the subcutaneous fossa of the left ischium. The preliminary clinical diagnosis was a vulvar mass.

#### 3.2.2 Imaging examinations

**Figure 2:**
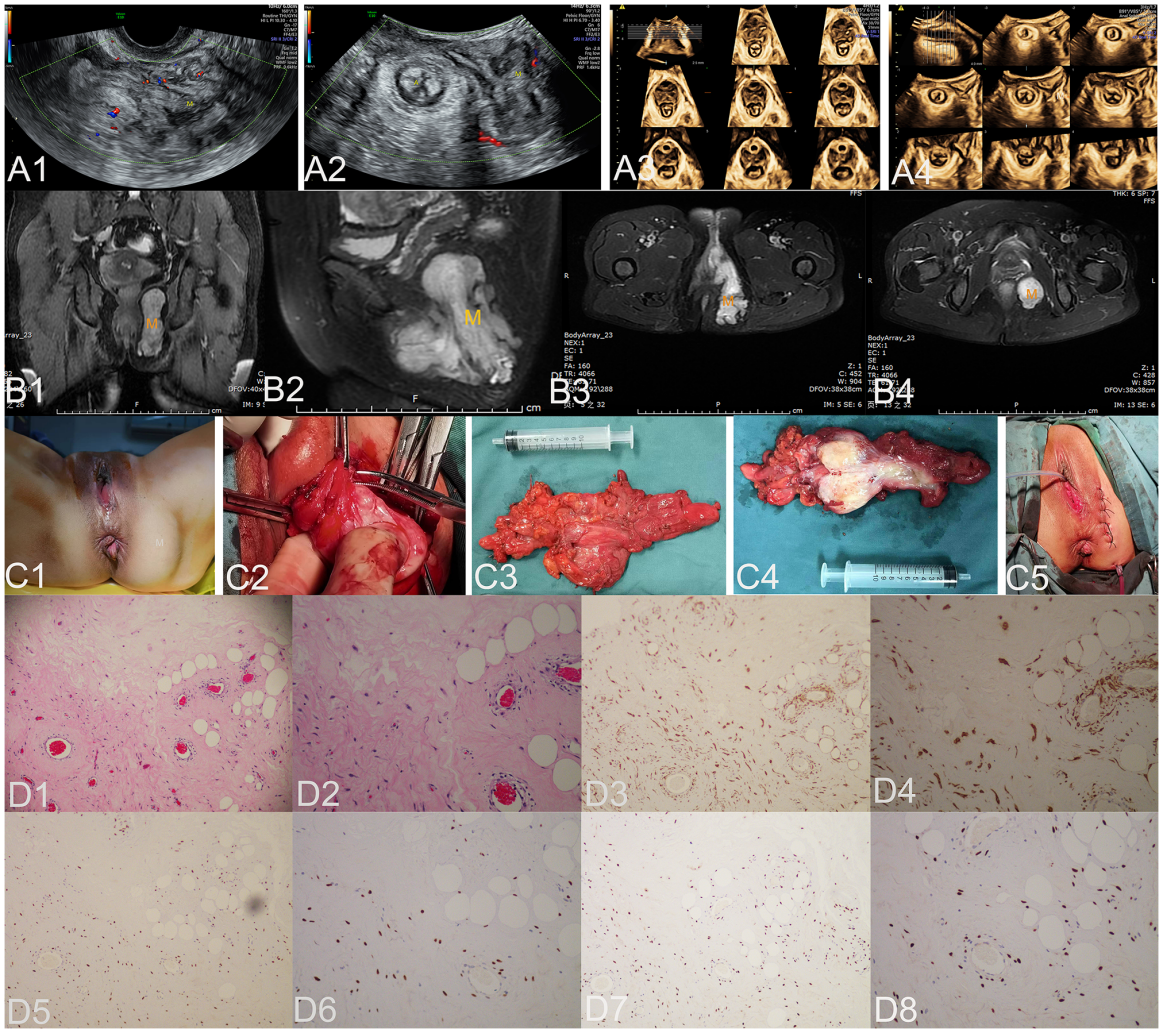
The ultrasound **(A1–A4)**, MRI **(B1–B4)**, surgical **(C1–C5)**, and pathological images **(D1–D8)** of case 2. US: In panel **(A1)**, a sagittal section of the left vulva obtained by transvaginal ultrasound reveals an irregular and uneven echo in the subcutaneous vulvar soft tissue, indicating the presence of an AAM lesion. The boundary is indistinct with no apparent capsule, extending upward below the HLAM plane and reaching 0.5 cm into the subcutaneous perineum (M represents AAM lesion). Panels **(A2)** and **(A4)** depict axial planes of the anus, while TUI axial planes of the anus are reconstructed by three-dimensional ultrasound. These images reveal that the lesion is located in the left ischiorectal fossa. The inner boundary was close to the left external anal sphincter, the left superficial perineal transverse muscle, and the left anal levator muscle. The outer boundary reached the pelvic wall (M represents the AAM lesion). Panel **(A3)** displays the TUI axial planes of HLAM reconstructed by three-dimensional ultrasound, demonstrating that the lesion does not extend to the anal levator plane. Panels **(A1)** and **(A2)** display the blood flow signals within the lesion. MRI: Panels **(B1–B4)** illustrate the pelvic coronal plane, the left sagittal plane of the pelvis, the axial plane of the high pelvic floor, and the axial plane of the low pelvic floor, respectively. These images reveal a prolonged T2 signal in the left perineum, ischiorectal fossa, and pelvic space, which define the boundaries of the lesions (M represents the AAM lesion), including their extent on both sides (left and right), upper and lower limits, and anterior and posterior extents. Surgery: Panel **(C1)** illustrates the lesion as a cystic solid mass measuring 6.0 × 5.0 cm, which is palpable in the subcutaneous fossa of the left ischium. Panel **(C2)** illustrates an irregular solid mass with multiple pseudopodia-like extensions infiltrating the adipose tissue in an extreme manner. The lesion, completely resected and displayed in panel **(C3)**, is a soft tissue mass measuring 12.0 × 10.0 × 3.0 cm. It exhibits an extremely irregular solid mass with multiple pseudopodia-like projections and a pink appearance. Panel **(C4)** displays the lesion upon sectioning, which appears as a gray color with medium soft tissue consistency. Panel **(C5)** illustrates the appearance of the vulva after the procedure is completed. HE: Under low magnification, panel **(D1)** shows a homogeneous distribution of spindle cells, blood vessels, and adipose tissue with varying diameters in the stroma of myxoid transformation (HE, ×100). Under high magnification in panel **(D2)**, the tumor cells were irregularly dispersed within the mucous stroma and infiltrated into the adipose tissue. These tumor cells exhibited a short fusiform and oval-shaped morphology without evident nuclear division, displaying mild characteristics (HE, ×200). IHC: Tumor cells exhibited strong positive staining for desmin in panel D3 (×100) and panel **(D4)** (×200), estrogen receptors in panel D5 (×100) and panel **(D6)** (×200), and progesterone receptors in panel **(D7)** (×100) and panel **(D8)** (×200). US, ultrasonography; MRI, magnetic resonance imaging; M, mass; A, anus; HLAM, hiatus of the levator ani muscle; HE, histopathological examination; IHC, immunohistochemistry; AAM, aggressive angiomyxoma; TUI, tomographic ultrasound imaging.

The transvaginal and transperineal pelvic floor ultrasound showed an extremely irregular and uneven echo mass measuring 15.8 × 4.9 × 5.4 cm in the left ischiorectal fossa (**Figures 2A1–4**). The boundary was unclear, with no obvious capsule present. It extended upward below the plane of the hiatus of the levator ani muscle, and the inner boundary was close to the left external anal sphincter, left superficial perineal transverse muscle, and left anal levator muscle. The inferior margin reached a depth of 0.5 cm subcutaneously in the perineum. There were numerous corded high echoes, striped blood flow signals, and an RI of 0.74. The ultrasonographic diagnosis suggested the presence of a solid heterogeneous soft tissue tumor in the left perineum, ischiorectal fossa, and pelvic space, which could possibly be an AAM. The pelvic MRI (including a pelvic plain scan, perfusion imaging, diffusion, and multi-directional delay enhancement) revealed prolonged T1 and T2 signals in the left perineum, ischiorectal fossa, and pelvic space. The maximum dimensions measured approximately 3.4 × 10.0 × 9.6 cm (left–right diameter × anterior–posterior diameter × superior–inferior diameter). Inhomogeneous enhancement was observed with evident diffusion restriction on diffusion-weighted imaging (DWI) (**Figures 2B1–4**). MRI revealed an abnormal signal in the left perineum, ischiorectal fossa, and pelvic space, differentiating the tumor from infection.

#### 3.2.3 Surgical findings

During surgery, a 6.0 × 5.0 cm-sized cystic solid mass can be observed and palpated in the subcutaneous fossa of the left ischium, which was soft and non-tender and did not affect vaginal patency (**Figure 2C1**). A longitudinal incision of 7.0 cm was made on the lateral aspect of the left perineum, cutting through the perineal skin and subcutaneous fat layer by layer. It was observed that the left ischiorectal fossa was significantly enlarged with a non-mobile mass measuring approximately 12.0 × 10.0 × 3.0 cm. The edge of the lesion appeared extremely irregular with multiple pseudopodia-like extensions into the adipose space (**Figure 2C2**). The blunt and sharp dissection proceeded outward along the left side of the mass, up to the left anal levator muscle, outward to the left obturator muscle, inward to the left pubic rectal muscle, and deep into the left ischiorectal fossa. The mass was completely dissected, the perineal deep transverse muscle and superficial transverse muscle were partially ruptured during dissection, and the rectal mucosa was smooth on digital rectal examination. The excised tissue, measuring 12.0 × 10.0 × 3.0 cm and displaying an extremely irregular shape with multiple pseudopodia-like projections and a pink appearance (**Figure 2C3**), exhibited a small amount of mucus and no obvious capsule upon sectioning (**Figure 2C4**) and was subsequently sent for pathological examination. The vulva returned to its normal appearance following the surgical procedure (**Figure 2C5**).

#### 3.2.4 Pathological examination results

Histopathological examination of the specimen revealed a large number of thick-walled and thin-walled blood vessels in the tumor tissue, which were surrounded by oval and short fusiform cells. Cell atypia was not evident, and a small amount of collagen fibers and mucus were observed between the cells (**Figures 2D1,2**). Immunohistochemical analysis of the specimens showed strong positive staining for desmin (**Figures 2D3,4**), vimentin, ER (**Figures 2D5,6**), and PR (**Figures 2D7,8**); RB1 also exhibited strong positive staining. CD34 was expressed in small amounts; CD31 showed positive expression within blood vessels; CDK4 had scattered positivity; P16 showed mild positivity; MDM2 displayed partial positivity. SMA, S-100, and pan-cytokeratin exhibited negative staining. The Ki-67 immunohistochemical index was approximately 2%. Molecular testing shows MDM2 negativity. The final histopathological diagnosis was AAM with positive surgical margins.

#### 3.2.5 Follow−up and outcomes

The patient reported the absence of postoperative discomfort, and no recurrence was observed during the 3-month and 8-month telephone follow-up assessments.

### 3.3 Case 3

#### 3.3.1 Clinical history

The patient, a 47-year-old woman, reported no specific discomfort and incidentally discovered a vulvar mass 6 months ago. She did not experience fever, abdominal pain, or vulvar pain. Regular evaluations were conducted at other medical facilities, which revealed a gradual increase in size over the past week. This led to the patient seeking treatment at our hospital. During the gynecological examination, a 4.0 × 3.0 cm mass was found in the right vulva with no tenderness and normal vaginal patency. The preliminary clinical diagnosis was a vulvar mass.

#### 3.3.2 Imaging examinations

**Figure 3:**
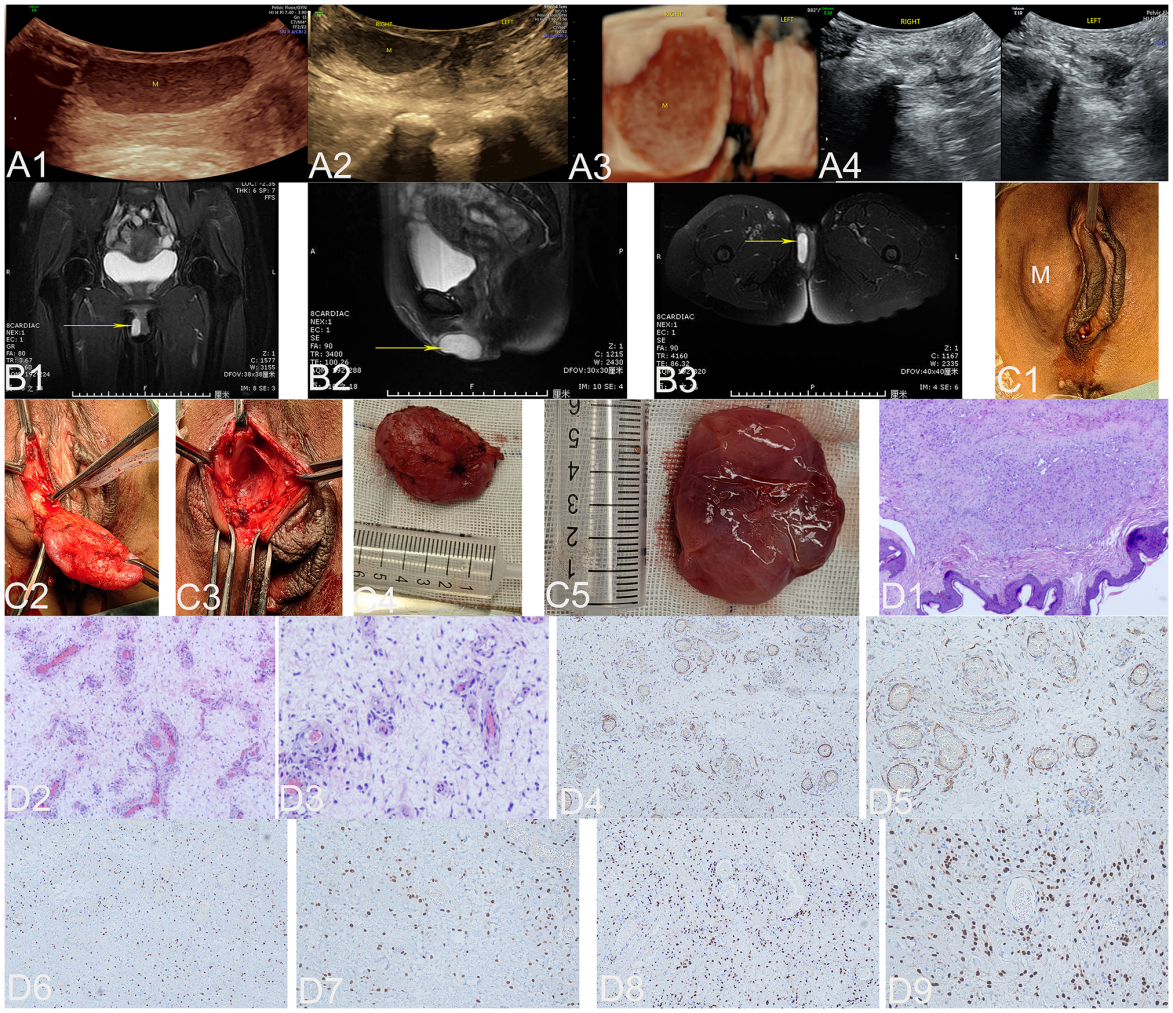
The ultrasound **(A1–A4)**, MRI **(B1–B3)**, surgical **(C1–C5)**, and pathological images **(D1–D9)** of case 3. US: The ultrasonic images in panels **(A1–A3)** depict the two-dimensional sagittal section of the right labia majora, the two-dimensional axial section, and the three-dimensional coronal section of both labia majora. These images clearly demonstrate a well-defined, thick-walled mass measuring 3.7 × 1.1 × 2.9 cm located between 0.3 cm and 1.4 cm beneath the skin of the upper two-thirds of the right labia majora (M represents an AAM lesion). The echogenicity of this mass varies from echo-poor to hypoechoic, with dense internal dot echoes and enhanced far-field echoes. Panel **(A4)** shows the sagittal section of the left and right labia majora 3 months after the operation, and no lesions were found. MRI: Panels **(B1–B3)** illustrate the pelvic coronal plane, the right sagittal plane of the pelvis, and the axial plane of the low pelvic floor, respectively. These images reveal a well-defined, round lesion with prolonged T1 and T2 signals in the right vulva (yellow arrow indicates the AAM lesion). Surgery: Panel **(C1)** illustrates a 4.0 × 3.0 cm mass located in the right labia majora (M represents the AAM lesion). Panel **(C2)** depicts an oval soft tissue mass, which is a lesion located on the skin of the right labia majora. Panel **(C3)** illustrates the cavity formed by the subcutaneous expansion and growth of the lesion. The lesion, completely resected and displayed in Panel **(C4)**, is an oval soft tissue mass measuring 4.0 × 3.0 cm with a pink appearance. Panel **(C5)** displays the lesion as pink, soft mucous tissue upon sectioning. HE: At low magnification, panel **(D1)** shows a tumor located within the dermis. The tumor has indistinct boundaries and is composed of spindle cells, blood vessels of varying sizes, and a myxoid matrix (HE, ×40). At low magnification in panel **(D2)**, an abundance of blood vessels can be observed within the backdrop of myxedema, ranging from noticeably slender-walled vessels to substantially thick-walled ones (HE, ×100). Upon high magnification, panel **(D3)** reveals neoplastic cells residing in the stroma of myxedema with reduced cellular density. These cells are characterized by abbreviated fusiform shape and occasional presence of atypical nuclei (HE, ×200). IHC: Tumor cells exhibited strong positive staining for desmin in panel **(D4)** (×100) and panel **(D5)** (×200), estrogen receptors in panel **(D6)** (×100) and panel **(D7)** (×200), and progesterone receptors in panel **(D8)** (×100) and panel **(D9)** (×200). US, ultrasonography; MRI, magnetic resonance imaging; M, mass; A, anus; HE, histopathological examination; IHC, immunohistochemistry; AAM, aggressive angiomyxoma.

The transperineal pelvic floor ultrasound showed a well-defined, thick-walled mass measuring 3.7 × 1.1 × 2.9 cm located between 0.3 cm and 1.4 cm beneath the skin of the upper two-thirds of the right labia majora. The mass exhibited echogenicity ranging from echo-poor to hypoechoic with internal dense dot echoes and enhanced far-field echoes (**Figures 3A1–4**). No peripheral or internal blood flow signals were detected. Based on the ultrasound findings, it was suggested that the abnormal subcutaneous echo observed in the right labia majora could indicate either a lipoma or a mucinous cyst. The MRI revealed a well-defined, round lesion with prolonged T1 and T2 signals in the right vulva, which was diagnosed as a vestibular gland cyst (**Figures 3B1–3**).

#### 3.3.3 Surgical findings

During the operation, a mass measuring 4.0 × 3.0 cm was identified in the right labia majora (**Figure 3C1**). A precise longitudinal incision measuring 2.5 cm was made on the surface of the mass, followed by careful blunt dissection to separate it (**Figure 3C2**). A cavity was formed by subcutaneous expansion and lesion growth (**Figure 3C3**). The resected lesion, measuring 4.0 × 3.0 cm and displaying an oval shape with a pink appearance (**Figure 3C4**), exhibited pink mucous soft tissue upon sectioning (**Figure 3C5**) and was subsequently sent for pathological examination.

#### 3.3.4 Pathological examination results

Histopathological examination of the specimen revealed that the tumor was located in the dermis with unclear boundaries and consisted of fusiform cells, blood vessels of different sizes, and a myxoid matrix (**Figures 3D1–3**). Immunohistochemical analysis demonstrated strong positive staining for desmin (**Figures 3D4,5**), ER (**Figures 3D6,7**), and PR (**Figures 3D8,9**), as well as caldesmon and vimentin expression. CD34 showed mild positivity, caldesmon showed weak positivity, S-100 showed scattered positivity, and β-catenin staining was positive in the plasma membrane. SMA, SOX10, STAT6, and ALK-IA4 expression were negative. The Ki-67 immunohistochemical index was estimated to be approximately 2%. The final histopathological diagnosis was AAM with positive surgical margins.

#### 3.3.5 Follow−up and outcomes

The patient reported no postoperative discomfort, and a postoperative ultrasound examination of the pelvic floor revealed no lesions. Therefore, there was no evidence of recurrence during the 1-month and 3-month follow-up visits.

### 3.4 Diagnostic accuracy of imaging

The overall diagnostic accuracy of ultrasound in locating and determining the extent of the lesion was 100% (3/3), but its specific diagnostic accuracy for identifying the pathological type of the lesion was 33% (1/3). However, MRI had a diagnostic accuracy of 100% (2/2) in locating and determining the extent of lesions, but its specific diagnostic accuracy for identifying pathological types of lesions was 0% (0/2).

## 4 Discussion

### 4.1 Clinical characteristics

AAM is a rare mesenchymal tumor that typically presents as a slow-growing soft tissue mass, occurring almost exclusively in the deep soft tissues of the vulvovaginal region, perineum, buttock, or pelvis of adult women. The reported age at presentation ranges from 9 months to 89 years, with the highest incidence observed during the fourth and fifth decades of life (6, 7). All three patients in this study were premenopausal women.

AAM lesions in women were primarily located in the vagina, vulva, perineal region, and extraperitoneal spaces of the pelvis (including the prevesical or retropubic, paravesical, paravaginal, pararectal, presacral, urethrovaginal, and rectovaginal spaces and the ischioanal fossa), with a wide range of involvement (8). Patients often visit the hospital due to a palpable, slow-growing, painless soft tissue mass in the vulvovaginal or perianal region or incidental findings of pelvic imaging without other discomfort. Some patients may experience symptoms related to local mass effects, such as dysuria, vaginal discomfort, or dyspareunia (9–15). Two out of the three cases included in this study presented with a gradually expanding and painless vulvar mass, while one exhibited significant anterior vaginal wall prolapse accompanied by urinary incontinence and dysuria. The initial clinical impressions may resemble other benign conditions like vestibular gland cysts, prolapse of the vaginal wall, obturator hernias, pelvic inflammatory masses, and other perineal tumors. The initial clinical diagnosis of the three cases in this study was identified as anterior vaginal wall prolapse, vulvar mass, and vulvar mass.

### 4.2 Imaging diagnosis

An accurate preoperative diagnosis should alert the surgeon to the necessity of wide excision, which is crucial for preventing local recurrence (5). The preoperative clinical manifestations are non-specific, and imaging examinations play an important role in the preoperative diagnosis, including determining the nature and extent of the suspected lesion. Imaging techniques including ultrasonography, computed tomography (CT), and MRI can reveal characteristic features of typical AAM, such as a multilocular or polypoid mass extending across the pelvic diaphragm with a pelvic and perineal component, along with finger-like projections infiltrating the surrounding soft tissues without invasion. These imaging modalities also showed laminated or swirling patterns (16–20).

On ultrasonography, a typical AAM presents as an ill-defined hypoechoic or isoechoic solid mass that extends down the lateral pelvis. It is separated from the pelvic organs and has an irregular and multi-lobed morphology, with finger-like or lingual-like extensions to the surrounding soft tissue or vulvar surface. The mass pushes against adjacent structures or protrudes from the vulvar surface, showing a laminated or swirled appearance of inner echogenicity due to alternating high and low echo intensities. Color Doppler imaging reveals abundant blood flow signals within and surrounding the tumor, while pulse Doppler imaging can detect arterial bloodstream spectrum. The obvious deformation of the lesion can be observed through probe pressure in real-time ultrasonography (21–24). In our study, one case occurred in the left rectal fossa and vulva with the typical ultrasound features described above, and AAM was accurately diagnosed by the sonographer. However, in the remaining two cases, the lesions were located in the urethrovaginal space and beneath the skin of the upper two-thirds of the right labia majora. Ultrasonographically, these lesions presented as oval hypoechoic to anechoic masses with well-defined margins, homogeneous distribution of coarse blip echoes, and no detectable intratumoral blood flow signal. The ultrasonic misdiagnosis was either a vaginal leiomyoma or a vulvar subcutaneous myxoma.

A typical AAM lesion is characterized by a well-defined mass that displaces adjacent structures on both CT and MRI. It exhibits hypoattenuation relative to the muscle on CT, hyperintense signal on T2-weighted MRI sequences, and hypointensity or isointensity on T1-weighted MRI sequences. Furthermore, both CT and MRI images exhibit avid and heterogeneous enhancement following intravenous contrast administration, potentially revealing a laminated or swirled appearance due to alternating high and low intensities (8, 17, 20, 24, 25). Two studies have documented the rare imaging characteristics of AAM, which include the presence of internal cystic degeneration, restricted diffusion, hemorrhage, and infiltration into adjacent organs (19, 24). However, due to its highly diverse morphological spectrum, AAM exhibits various imaging features, posing challenges for preoperative imaging diagnosis. Among our three cases, two patients underwent pelvic MRI. One patient exhibited prolonged T1 and T2 signals with inhomogeneous enhancement and evident diffusion restriction on DWI, suggesting a tumor or infection. The other patient presented a well-defined, round lesion with prolonged T1 and T2 signals in the right vulva, suggestive of a vestibular gland cyst.

### 4.3 Pathological characteristics

#### 4.3.1 Gross pathology

During the operation, on gross examination, typical AAM is usually unencapsulated and poorly or vaguely circumscribed. It may blend imperceptibly with the surrounding soft tissue, extending like fingers or tongues into the adjacent soft tissue or vulvar surface. Most of the completely resected specimens were found to be anomalous and multilobed (14, 26, 27). In our study, the lesion in one case was located in the ischiorectal fossa and vulva, showing the typical growth pattern and shape described above. The lesions of the other two cases were oval with a clear envelope; they expanded into hemispherical protrusions within the vaginal cavity and on the surface of the labia majora. The tumor size varied, ranging from 1.5 to 60 cm in the greatest dimension (28). The maximum diameters of the three lesions in this study were 5 cm, 12 cm, and 4 cm. During sectioning, a typical AAM exhibits a tan-pink to tan-gray coloration. It presents as a voluminous mass with a resilient texture and displays a rubbery, lustrous, gelatinous appearance that is exclusively myxoid, fibro-myxoid, or predominantly fibrosclerotic (1, 26, 27, 29). Areas of congested blood vessels of various sizes, hemorrhage, small-to-large cystic foci, and fibrous tissue in the cord may be present (24, 26, 29). In our study, the incisions of two regular lesions showed small cystic foci in exclusively myxoid tissue ranging from tan-pink to grayish-brown, while the incision of the highly irregular lesion exhibited small cystic foci and cord-like fibrous tissue within the grayish-brown fibro-myxoid tissue.

#### 4.3.2 Histopathological and immunohistochemical pathology

The combination of histopathological examination and immunohistochemical analysis can effectively establish a definitive diagnosis for AAM. The typical histological features of AAM include non-encapsulated lesions composed of small fusiform to stellate cells embedded in a loose mucoid matrix. This matrix contains a substantial number of small and medium blood vessels with varying wall thicknesses (1, 2, 4, 13, 15, 26, 27, 30, 31). On microscopic examination, all specimens from our three cases exhibited the typical features of AAM described above. Immunohistochemically, the tumor cells tested positive for desmin, vimentin, SMA, and CD34 and negative for S-100 and CK. The Ki-67 index was approximately 1%–3%. In most female patients, these characteristics were accompanied by strongly positive expression of ER and PR (15, 31–34). Immunohistochemically, the tumor cells in our three cases exhibited strong positive staining for desmin, vimentin, ER, and PR. SMA showed strong positivity in case 1 but was negative in cases 2 and 3. CD34 was negative in case 1, displayed a small amount of positive staining in case 2, and exhibited mild positivity in case 3. S-100 demonstrated partial positivity in case 1, was negative in case 2, and showed scattered positivity in case 3. The Ki-67 immunohistochemical indices for our three cases were approximately 1%, 2%, and 2%. During molecular testing, the rearrangement of the HMGA2 gene is commonly observed in vulvovaginal AAM, and it serves as a valuable diagnostic tool. This is because most mesenchymal lesions that closely resemble this condition exhibit negative results for this gene rearrangement, although some other mesenchymal lesions such as leiomyomas at this site may show positive results (35, 36). Two out of our three cases underwent molecular testing, one of which exhibited rearrangement in the HMGA2 gene. Additionally, our study identified other molecular markers such as CTNNB1, APC gene mutation, and MDM2 for differential diagnosis.

### 4.4 Follow-up and outcomes

AAM is characterized by a high recurrence rate of 36%–72% after surgery. Wide surgical excision is considered the gold standard treatment for AAM, and there is no universal consensus regarding hormonal therapy. Most studies (85%) describe successful surgical excision followed by either clinical or radiological (ultrasound or MRI) follow-up (37). The lesions in all three cases included in our study were completely resected, and there was no evidence of recurrence based on clinical and ultrasonographic follow-up for cases 1 and 3 at the 7-month and 3-month visits, respectively. For case 2, no recurrence was observed during the telephone follow-ups at both the 3-month and 8-month marks, with the patient instructed to self-assess.

## 5 Conclusions

In conclusion, our findings indicate that due to the atypical clinical manifestations, imaging features, and gross appearance of some AAM, the presence of lesions in various extraperitoneal spaces of the pelvis (including the vagina, vulva, perineal region, prevesical or retropubic space, paravesical space, paravaginal space, pararectal space, presacral space, urethrovaginal space rectovaginal spaces, and ischioanal fossa), particularly when exhibiting extensive abdominal/perineal extension in reproductive-age female patients during the fourth to fifth decade, should raise suspicion for AAM. Even if a vulvovaginal lesion presents with a superficial location, small size, limited scope, and regular shape, suspicion of atypical AAM should arise when palpation reveals toughness, tensility, and deformability under pressure. US reveals a well-defined hypoechoic to anechoic mass with uniformly distributed coarse dot echoes, with or without detectable intratumoral blood flow signal. MRI shows prolonged T1 and T2 signals with inhomogeneous enhancement and evident diffusion restriction on DWI.

## Data availability statement

The original contributions presented in the study are included in the article/supplementary material. Further inquiries can be directed to the corresponding author.

## Ethics statement

The studies involving humans were approved by Medical Ethics Committee of Tongji Hospital Affiliated to Tongji Medical College of Huazhong University of Science and Technology (approval No. TJ-IRB20230834). The studies were conducted in accordance with the local legislation and institutional requirements. The participants provided their written informed consent to participate in this study. Written informed consent was obtained from the individual(s) for the publication of any potentially identifiable images or data included in this article.

## Author contributions

LZ: Conceptualization, Data curation, Formal Analysis, Investigation, Methodology, Project administration, Resources, Software, Supervision, Writing – original draft, Writing – review & editing, Validation, Visualization. RL: Conceptualization, Data curation, Investigation, Methodology, Project administration, Resources, Supervision, Validation, Visualization, Writing – review & editing. JP: Investigation, Resources, Validation, Writing – review & editing.
