# Corrigendum: Misleading clinical and imaging features in atypical aggressive angiomyxoma of the female vulvovaginal or perianal region: report of three cases and review of the literature

**DOI:** 10.3389/fonc.2024.1406876

**Published:** 2024-04-12

**Authors:** Ling Zhang, Rong Liu, Jian Peng

**Affiliations:** ^1^Department of Obstetrics and Gynecology, National Clinical Research Center for Obstetrics and Gynecology, Tongji Hospital, Tongji Medical College, Huazhong University of Science and Technology, Wuhan, China; ^2^Key Laboratory of Cancer Invasion and Metastasis (Ministry of Education), Hubei Key Laboratory of Tumor Invasion and Metastasis, Tongji Hospital, Tongji Medical College, Huazhong University of Science and Technology, Wuhan, China; ^3^Department of Radiology, Tongji Hospital, Tongji Medical College, Huazhong University of Science and Technology, Wuhan, China

**Keywords:** aggressive angiomyxoma, perineal soft tissue mass, vestibular gland cyst, vaginal wall prolapse, ultrasonography, magnetic resonance imaging

In the published article, there was an error in **Figure 1** as published. The presence of **Figure 1B2** can be attributed to an oversight on the part of the authors, who mistakenly included the wrong image. The corrected **Figure 1** and its caption **Figure 1** "The ultrasound **(A1–A10)**, surgical **(B1–B5)**, and pathological images **(C1–C8)** of case 1. US: panels **(A1–A3)** respectively display the median sagittal sections of the transperineal ultrasound in the resting state, maximum contraction state, and maximum Valsalva state of the patient in the lithotomy position preoperatively. In panel **(A1)**, an AAM lesion is observed within the urethrovaginal space; it ascends to the posterior aspect of the bladder in panel **(A2)** and descends to the vaginal opening in panel **(A3)** (M represents the AAM lesion). All the lesions appeared as homogeneous hypoechoic masses with well-defined margins. Panels **(A4, A5)** show the median sagittal sections of perineal ultrasound in the resting state and maximum Valsalva state, respectively, of the patient 7 months post-surgery without any observed lesions. Panels **(A6, A8)** display the axial planes of HLAM reconstructed by three-dimensional ultrasound in the resting state preoperatively and during the Valsalva maneuver. Panel **(A7)** displays the axial plane of TUI during the maximum contraction state preoperatively. These axial planes provide information about the size, shape, and location of the AAM lesions. In panel **(A6)**, the lesion is observed protruding posteriorly from behind the urethra toward the vagina; in panel **(A7)**, the lesion has ascended completely above the HLAM; in panel **(A8)**, during the Valsalva maneuver, the lesion descends through the HLAM (M represents an AAM lesion). Panels **(A9, A10)** demonstrate the axial plane of the HLAM in the resting state and maximum Valsalva state, respectively, 7 months post-surgery for patient without any observed lesions. Surgery: panels **(B1, B2)** depict the lesion as an oval-shaped mass protruding from the anterior vaginal wall into the vaginal cavity. Panel **(B3)** displays the completely resected lesion, which is a soft tissue mass measuring 4.0 × 5.0 cm and exhibiting an oval shape with a pink appearance. Panel **(B4)** displays the lesion upon sectioning, which appears as a gray color with medium soft tissue consistency. Panel **(B5)** depicts the appearance of the vulva after the procedure has been completed. HE: Panel **(C1)**, at low magnification, shows that the tumor boundary appears indistinct, revealing a myxedema matrix interspersed with thin collagen fibers and blood vessels of varying thickness within the tumor. The tumor exhibits areas of loose and dense cellularity, with spindle cells uniformly scattered in dense regions without apparent organization (HE, ×100). As shown in panel **(C2)**, at high magnification, the tumor cells exhibited a low density and assumed a stellate or fusiform morphology without evident pleomorphism (HE, ×200). IHC: Tumor cells exhibited strong positive staining for desmin in panel **(C3)** (×100) and panel **(C4)** (×200), estrogen receptors in panel **(C5)** (×100) and panel **(C6)** (×200), and progesterone receptors in panel **(C7)** (×100) and panel **(C8)** (×200). US, ultrasonography; U, urethra; M, mass; V, vagina; A, anus; HLAM, hiatus of levator ani muscle; HE, histopathological examination; IHC, immunohistochemistry; AAM, aggressive angiomyxoma; TUI, tomographic ultrasound imaging." appear below.

**Figure 1:**
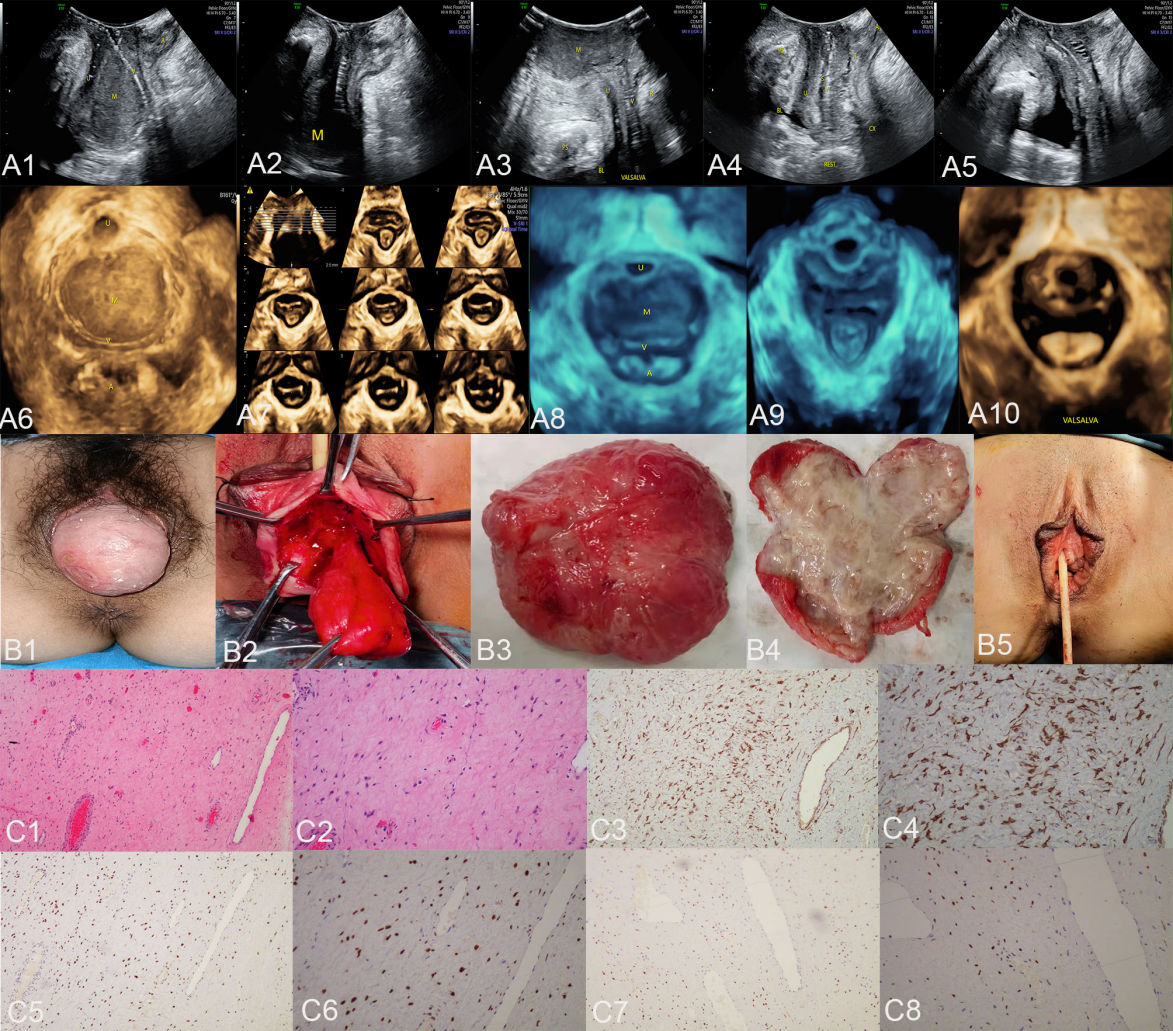
The ultrasound **(A1–A10)**, surgical **(B1–B5)**, and pathological images **(C1–C8)** of case 1. US: panels **(A1–A3)** respectively display the median sagittal sections of the transperineal ultrasound in the resting state, maximum contraction state, and maximum Valsalva state of the patient in the lithotomy position preoperatively. In panel **(A1)**, an AAM lesion is observed within the urethrovaginal space; it ascends to the posterior aspect of the bladder in panel **(A2)** and descends to the vaginal opening in panel **(A3)** (M represents the AAM lesion). All the lesions appeared as homogeneous hypoechoic masses with well-defined margins. Panels **(A4, A5)** show the median sagittal sections of perineal ultrasound in the resting state and maximum Valsalva state, respectively, of the patient 7 months post-surgery without any observed lesions. Panels **(A6, A8)** display the axial planes of HLAM reconstructed by three-dimensional ultrasound in the resting state preoperatively and during the Valsalva maneuver. Panel **(A7)** displays the axial plane of TUI during the maximum contraction state preoperatively. These axial planes provide information about the size, shape, and location of the AAM lesions. In panel **(A6)**, the lesion is observed protruding posteriorly from behind the urethra toward the vagina; in panel **(A7)**, the lesion has ascended completely above the HLAM; in panel **(A8)**, during the Valsalva maneuver, the lesion descends through the HLAM (M represents an AAM lesion). Panels **(A9, A10)** demonstrate the axial plane of the HLAM in the resting state and maximum Valsalva state, respectively, 7 months post-surgery for patient without any observed lesions. Surgery: panels **(B1, B2)** depict the lesion as an oval-shaped mass protruding from the anterior vaginal wall into the vaginal cavity. Panel **(B3)** displays the completely resected lesion, which is a soft tissue mass measuring 4.0 × 5.0 cm and exhibiting an oval shape with a pink appearance. Panel **(B4)** displays the lesion upon sectioning, which appears as a gray color with medium soft tissue consistency. Panel **(B5)** depicts the appearance of the vulva after the procedure has been completed. HE: Panel **(C1)**, at low magnification, shows that the tumor boundary appears indistinct, revealing a myxedema matrix interspersed with thin collagen fibers and blood vessels of varying thickness within the tumor. The tumor exhibits areas of loose and dense cellularity, with spindle cells uniformly scattered in dense regions without apparent organization (HE, ×100). As shown in panel **(C2)**, at high magnification, the tumor cells exhibited a low density and assumed a stellate or fusiform morphology without evident pleomorphism (HE, ×200). IHC: Tumor cells exhibited strong positive staining for desmin in panel **(C3)** (×100) and panel **(C4)** (×200), estrogen receptors in panel **(C5)** (×100) and panel **(C6)** (×200), and progesterone receptors in panel **(C7)** (×100) and panel **(C8)** (×200). US, ultrasonography; U, urethra; M, mass; V, vagina; A, anus; HLAM, hiatus of levator ani muscle; HE, histopathological examination; IHC, immunohistochemistry; AAM, aggressive angiomyxoma; TUI, tomographic ultrasound imaging.

The authors apologize for this error and state that this does not change the scientific conclusions of the article in any way. The original article has been updated.

